# Protection of Pentoxifylline against Testis Injury Induced by Intermittent Hypobaric Hypoxia

**DOI:** 10.1155/2016/3406802

**Published:** 2016-08-25

**Authors:** Chen Yao, Gang Li, Yeyong Qian, Ming Cai, Hong Yin, Li Xiao, Wei Tang, Fengjie Guo, Bingyi Shi

**Affiliations:** ^1^Organ Transplant Institute, Chinese PLA 309th Hospital, Beijing 100091, China; ^2^Institute of Genetic Engineering, School of Basic Medical Sciences, Southern Medical University, Guangzhou 510515, China

## Abstract

To investigate the effect of pentoxifylline (PTX) on spermatogenesis dysfunction induced by intermittent hypobaric hypoxia (IHH) and unveil the underlying mechanism, experimental animals were assigned to Control, IHH+Vehicle, and IHH+PTX groups and exposed to 4 cycles of 96 h of hypobaric hypoxia followed by 96 h of normobaric normoxia for 32 days. PTX was administered for 32 days. Blood and tissue samples were collected 7 days thereafter. Serum malondialdehyde levels were used to assess lipid peroxidation; ferric-reducing antioxidant power (FRAP), superoxide dismutase, and catalase and glutathione peroxidase enzyme activities were assessed to determine antioxidant capacity in various samples. Testis histopathology was assessed after hematoxylin-eosin staining by Johnsen's testicular scoring system. Meanwhile, testosterone synthase and vimentin amounts were assessed by immunohistochemistry. Sperm count, motility, and density were assessed to determine epididymal sperm quality. IHH treatment induced significant pathological changes in testicular tissue and enhanced serum lipid peroxide levels, while reducing serum FRAP, antioxidant enzyme activities, and testosterone synthase expression. Moreover, IHH impaired epididymal sperm quality and vimentin structure in Sertoli cells. Oral administration of PTX improved the pathological changes in the testis. IHH may impair spermatogenesis function of testicular tissues by inducing oxidative stress, but this impairment could be attenuated by administration of PTX.

## 1. Introduction

Humans living at high altitudes are exposed to hypobaric hypoxia, a condition where reduced pressure decreases oxygen availability resulting in reduced oxygen levels [1, 2]. Several activities such as mountain sports, tourism, and jobs (i.e., customs agents, miners, and educational and healthcare workers) performed in areas over 1,500 meters above sea level (asl) involve intermittent exposure to hypobaric hypoxia (IHH) [3–5]. Chronic and IHH have been associated with increased oxidative damage in the testis [6]. IHH induces high levels of lipid peroxidation, reduces glutathione reductase activity, and decreases the epididymal sperm count [7]. This environment causes more severe damage in reproductive function to immigrants than natives. There is no difference in fertility rate between high-altitude natives and plain residents. However, the reproductive function of plain residents would be seriously injured once they move to altitudes. Similar facts have been discussed by several scientists. For instance, Verratti et al. reported that after 26 days of exposure to high altitude (2000 m to 5600 m), male healthy mountain trekkers living at sea level developed oligospermia, a reduction of the total number of motile sperms and an increase in abnormal or immature spermatozoa [8]. Gonzales also observed a reduction of reproductive activity in people recently migrated to Andes in Peru compared with natives [9].

IHH causes physiological changes in individuals living at high-altitude, who show hypoxemia due to the reduced oxygen pressure. Simultaneously, temperature variations, enhancement of UV exposure, and changes in metabolic rate result in increased reactive oxygen species (ROS) levels in high-altitude populations [10]. Excess ROS overperoxidize membrane lipids, damage the DNA and oxidize proteins, and influence the production of mitochondrial ATP. Oxidative stress also causes cell swelling, decreases cell membrane fluidity, prevents the maintenance of ionic gradients, and leads to tissue inflammation, which may result in structural and functional detrimental effects [10–12]. IHH treatment also causes pathological changes in the testicular tissue and decreases the epididymal sperm density by enhancing oxidative stress; this damage can be relieved by administration of antioxidants [6, 7, 13]. For example, polyunsaturated fatty acids improve the peroxidative state induced by IHH in plasma and testicular tissues by enhancing the total antioxidant ability [14].

Pentoxifylline (PTX) is a methylxanthine derivative with multiple hemorheologic properties. PTX, a nonselective phosphodiesterase inhibitor, inhibits the conversion of cAMP to AMP, increases cAMP levels, improves cell function and hemorheology, and possesses an angiectatic activity [15]. PTX through increasing intracellular cyclic AMP in red blood cells improves oxygen delivery to ischemic tissues, also increasing cyclic AMP amounts in polymorphonuclear leukocytes and decreasing oxygen-free radical production [16–19]. Glycolysis is the main metabolic pathway of energy supply under hypoxia. PTX can increase the mitochondrial glycolysis and respiratory rates, accelerate ATP production, and improve microcirculation [20]. Other studies indicated that PTX also inhibits conglutination, activating neutrophilic granulocytes, reducing blood viscosity, increasing partial pressure of oxygen and anti-inflammation, eliminating free radicals, inhibiting the expression of NF-*κ*B and TNF-*α* mRNA, and reducing cell apoptosis [21–24].

Based on the above findings, an animal model of spermatogenesis dysfunction was established by IHH. In the model animals, serum lipid peroxidative status (malondialdehyde [MDA] levels) and antioxidant activity (superoxide dismutase [SOD], catalase [CAT], and glutathione peroxidase [GPX]) were assessed, as well as pathological changes of the testicular tissue, spermatogenesis, and testosterone synthase amounts. Sertoli cells are critical in sperm production, and it was shown that reduced testosterone levels are coupled with decreased expression of steroidogenic acute regulatory protein (StAR) and 3*β*-hydroxysteroid dehydrogenase (3*β*-HSD) [25, 26]. Finally, PTX was administered to investigate its effects on spermatogenesis dysfunction induced by intermittent IHH.

## 2. Materials and Methods

### 2.1. Study Design

A total of 24 male 12-week-old Sprague-Dawley rats were obtained from the Animal Breeding Center at Chinese PLA 309th Hospital. The experiments were approved by the institutional animal care and subcommittee of Chinese PLA 309th Hospital. All animals were kept in a clean environment under a 12 h light/12 h dark cycle. After adaptation, the animals were divided into 3 groups; Control (PBS, p.o., qd, 2 mL/d, *n* = 8), IHH+Vehicle (PBS, p.o., qd, 2 mL/d, ~428 tor, PO_2_ 90 mmHg, *n* = 8), and PTX+IHH (PTX 0.3 g/kg p.o., qd, 2 mL/d, ~428 tor, PO_2_ 90 mmHg, *n* = 8) groups. All animals were exposed to 4 cycles of 96 h of hypobaric hypoxia followed by 96 h of normobaric normoxia for 32 days. PTX supplementation was maintained for 32 days in this study. Blood and tissue samples were collected 7 days thereafter.

### 2.2. Serum MDA Levels and Total Antioxidant Ability

Blood samples (1 mL) were collected by heart puncture, centrifuged for 30 min, aliquoted (100 *μ*L per tube), and stored at −80°C. Serum MDA amounts were determined using Lipid Peroxidation MDA Assay Kit (S0131, Beyotime, Shanghai, China) following the manufacturer instructions. Briefly, samples were diluted with phosphate buffered saline (1 : 5) and 800 *μ*L of trichloroacetic acid (TCA, 28% w/v) was added to 400 *μ*L of this mixture. After centrifugation at 3000 ×g for 30 min, the precipitate was dissolved in sulfuric acid, and 600 *μ*L of the resulting mixture was added to 150 *μ*L of 1% w/v 2-thiobarbituric acid (TBA). The mixture was then incubated for 15 min in a boiling water bath, followed by addition of 4 mL n-butanol. After centrifugation, absorbance was recorded in the supernatant at 532 nm on a UV-160-A Shimadzu double beam spectrophotometer (Japan).

Ferric-reducing antioxidant power (FRAP) was examined using T-AOC Assay Kit (S0116, Beyotime, Shanghai, China) following the product manual. Briefly, the medium was exposed to Fe^3+^ and the antioxidants present started to produce Fe^2+^. The FRAP reagent was prepared freshly and contained 25 mL of 300 mM acetate buffer (pH 3.6), 2.5 mL of 10 mM 2,4,6-tripyridyl-s-triazine (TPTZ) solution in 40 mM HCl, and 2.5 mL of 20 mM ferric chloride (FeCl_3_-6H_2_O). The blue complex formed by Fe^2+^ and TPTZ was quantified by measuring absorbance at 593 nm.

### 2.3. Antioxidant Enzyme Activity in Testes

SOD activity (S0101, Beyotime Biotechnology, Shanghai, China), CAT activity (S0051, Beyotime Biotechnology, Shanghai, China), and GPx activity (S0058, Beyotime Biotechnology, Shanghai, China) were determined using commercial kits following the product manuals, as previously described [27].

### 2.4. Testis Histopathology and Testosterone Synthase Levels

Testis histopathology was assessed by immunohistochemical (IHC) assay. Briefly, testicular tissue specimens were collected, fixed with Bouin's solution for 6 hours, and kept in 70% ethanol. After dehydration by graded ethanol series, the samples were cleared by xylene and paraffin embedded. Then, 4 *μ*m thick sections were dewaxed in xylene, rehydrated in serial graded ethanol solutions, and submitted to hematoxylin-eosin (H&E) staining and IHC. A total of 30 independent seminiferous tubule fields were evaluated in each sample, and the histopathological changes were evaluated by Johnsen's testicular scoring system; a score between 1 (very poor) and 10 (normal) was given to each tubule [28]. Testosterone synthase levels were examined by IHC assay. Tissue sections were incubated for 10 min in 0.3% hydrogen peroxide to block endogenous peroxidase activity before IHC. After washing with PBS, tissue sections were boiled in 0.01 M citrate buffer (pH 6.0) for 10 min. The sections were washed with PBS, incubated in 0.1% triton X-100 and 5% goat serum for 30 min, and followed by anti-StAR (Santa Cruz Biotechnology, USA, 1: 200) and anti-3*β*-HSD (Santa Cruz, 1: 200) primary antibodies overnight at 4°C in a humidified chamber. IHC was performed using the MaxVision HRP-Polymer immunohistochemistry kit (Maxim, China), and development was carried out with diaminobenzidine (DAB).

### 2.5. Epididymal Sperm Quality

Semen analysis was performed as previously described [25]. Spermatozoa were collected from bilateral caudal epididymis and incubated in 2 mL Ham's F-10 medium containing 0.5% bovine serum albumin. Five minutes later, the epididymal sperm count was determined using a hemocytometer. Sperm motility was analyzed under a light microscope (Leica, Germany) in 10 fields according to the 5th edition of World Health Organization (WHO) Laboratory Manual for the Examination and Processing of Human Semen (2010) [29]. Epididymal sperm density was calculated as the ratio of sperm count to caudal epididymis weight.

### 2.6. Statistical Analysis

The SPSS statistical software (version 11.0, IBM, USA) was used for statistical analyses. Data are mean ± standard deviations (SD). Differences among the three groups were analyzed by a nonparametric test (Kruskal-Wallis). Dual comparisons between groups were performed with Mann-Whitney *U*-test. *p* < 0.05 was considered statistically significant.

## 3. Results

### 3.1. Serum Lipid Peroxide Levels and Total Antioxidant Activity

**Figure 1:**
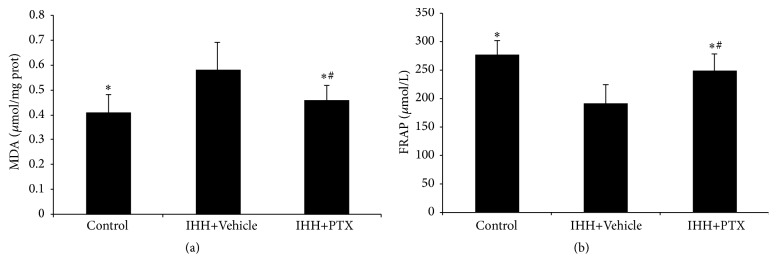
Effect of PTX on antioxidant capacity and lipid peroxidation in plasma. MDA levels (a) and FRAP levels (b) were assessed in plasma. Control group (*n* = 8); intermittent hypobaric hypoxia (IHH) group (*n* = 8); pentoxifylline (PTX)+IHH group (*n* = 8). Data were presented as mean ± standard deviations (SD). ^*∗*^ *p* < 0.05 compared with IHH+Vehicle. ^#^ *p* < 0.05 compared with Control.

After the IHH cycles, FRAP levels were lower compared with values obtained for the Control group (Control: 276.23 ± 24.76; 189.17 ± 33.54; and 247.82 ± 29.38) (*p* < 0.05). MDA amounts were 0.41 ± 0.07, 0.58 ± 0.11, and 0.46 ± 0.06 for Control, IHH+Vehicle, and PTX+IHH groups, respectively. MDA levels were therefore significantly higher in the IHH+Vehicle and PTX+IHH groups compared with the Control group (*p* < 0.05) (Figure 1).

### 3.2. Epididymal Sperm Density and Epididymal Sperm Mobility

**Figure 2:**
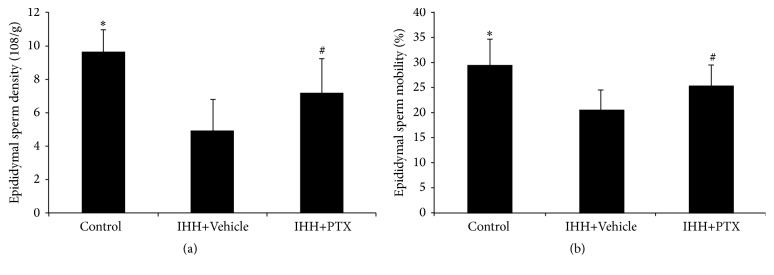
Effect of PTX on epididymal sperm parameters. Epididymal sperm density (a) and mobility (b) were analyzed under a light microscope. Control group (*n* = 8); intermittent hypobaric hypoxia (IHH) group (*n* = 8); pentoxifylline (PTX)+IHH group (*n* = 8). Data were presented as mean ± standard deviation (SD). ^*∗*^ *p* < 0.05 compared with IHH+Vehicle. ^#^ *p* < 0.05 compared with Control.

Epididymal sperm density and mobility were significantly reduced by IHH. Epididymal sperm densities (10^8^/g) were 9.64 ± 1.31, 4.95 ± 1.83, and 7.16 ± 2.06 for Control, IHH+Vehicle, and PTX+IHH, respectively; meanwhile, epididymal sperm mobility rates (%) were 29.54 ± 5.17, 20.66 ± 3.67, and 25.23 ± 4.12, respectively. There were significant differences between Control and IHH+Vehicle groups (*p* < 0.05) and Control and PTX+IHH group (*p* < 0.05). Interestingly, PTX treatment prevented the decline in sperm concentration and mobility (Figure 2).

### 3.3. Histopathological Changes in Testicular Tissue

**Figure 3:**
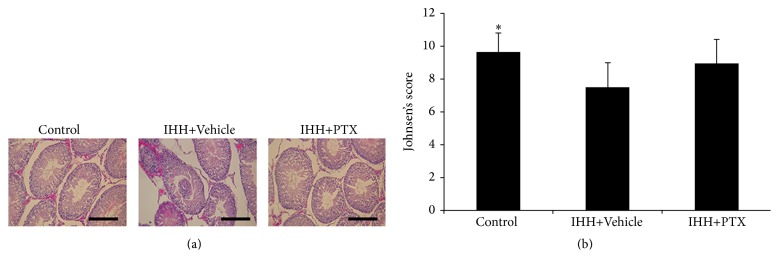
Effect of PTX on testes histopathology. (a) Representative micrographs of hematoxylin-eosin-stained sections in the testes of rats, 400x, insert bar = 100 *μ*m. (b) Quantification of Johnson's score of testes injuries. Control group (*n* = 8); intermittent hypobaric hypoxia (IHH) group (*n* = 8); pentoxifylline (PTX)+IHH group (*n* = 8). Data were presented as mean ± standard deviation (SD). ^*∗*^ *p* < 0.05 compared with IHH+Vehicle.

IHH induced significant histopathological changes, including disordered arrangement and exfoliation of seminiferous epithelium cells, and reduced Johnsen's scores. Johnsen's scores were 9.66 ± 1.17, 7.47 ± 1.52, and 8.93 ± 1.49 for Control, IHH+Vehicle, and PTX+IHH, respectively. There was a significant difference between the Control and IHH+Vehicle groups (*p* < 0.05). Importantly, administration of PTX improved the above indicators (Figure 3).

### 3.4. Activity of Antioxidant Enzymes in Testicular Tissue

**Figure 4:**
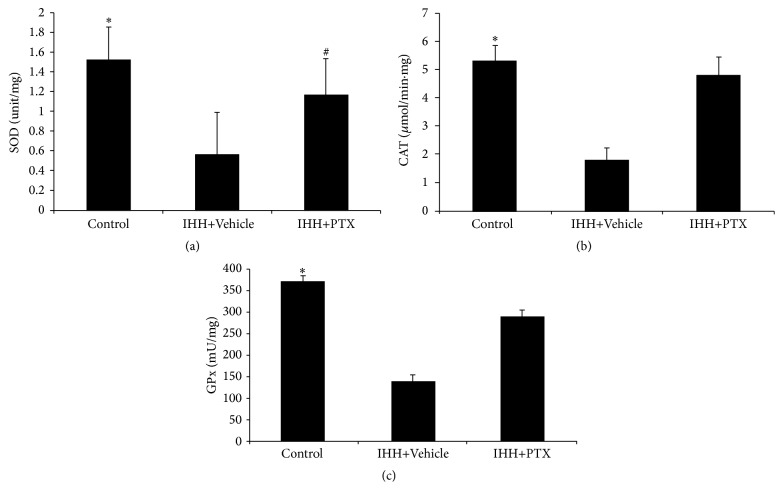
Effect of PTX on antioxidant enzymes activity in testis. Superoxide dismutase (SOD, a), catalase (CAT, b), and glutathione peroxidase (GPx, c) activities were assessed in testis of three groups. Control group (*n* = 8); intermittent hypobaric hypoxia (IHH) group (*n* = 8); pentoxifylline (PTX)+IHH group (*n* = 8). Data were presented as mean ± standard deviation (SD). ^*∗*^ *p* < 0.05 compared with IHH+Vehicle. ^#^ *p* < 0.05 compared with Control.

SOD activity levels were 1.51 ± 0.34, 0.56 ± 0.43, and 1.17 ± 0.36 units/mg for Control, IHH+Vehicle, and PTX+IHH, respectively. CAT activity levels were 5.32 ± 0.51, 1.79 ± 0.40, and 4.82 ± 0.62 *μ*mol/min*∗*mg for Control, IHH+Vehicle, and PTX+IHH, respectively, for GPX levels of 368.93 ± 11.74, 138.52 ± 16.31, and 289.47 ± 14.53 mU/mg, respectively. There were significant differences between Control and IHH+Vehicle group (*p* < 0.05) in all the above parameters. IHH treatment decreased the activity of antioxidant enzymes in the testicular tissue, including SOD, CAT, and GPX, an effect reversed by PTX (Figure 4).

### 3.5. Testosterone Synthase Levels

**Figure 5:**
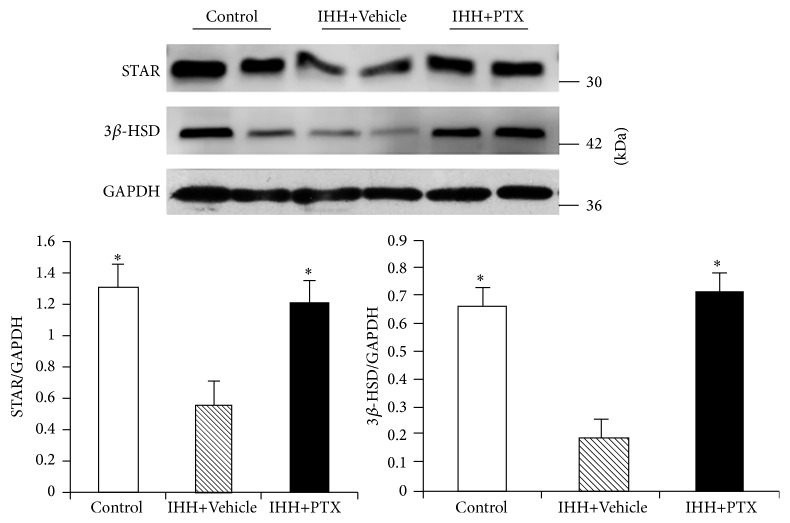
Effect of PTX on the expression of testosterone synthases 3*β*-HSD and StAR. Evaluation of protein expression by using WB. Control group (*n* = 8); intermittent hypobaric hypoxia (IHH) group (*n* = 8); pentoxifylline (PTX)+IHH group (*n* = 8). Data were presented as mean ± standard deviation (SD). ^*∗*^ *p* < 0.05 versus IHH+Vehicle.

The expression levels of the testosterone synthases 3*β*-HSD and StAR were significantly reduced in IHH conditions. However, PTX administration blocked the inhibition by IHH of 3*β*-HSD and StAR (Figure 5).

### 3.6. Morphological Changes in Sertoli Cells

**Figure 6:**
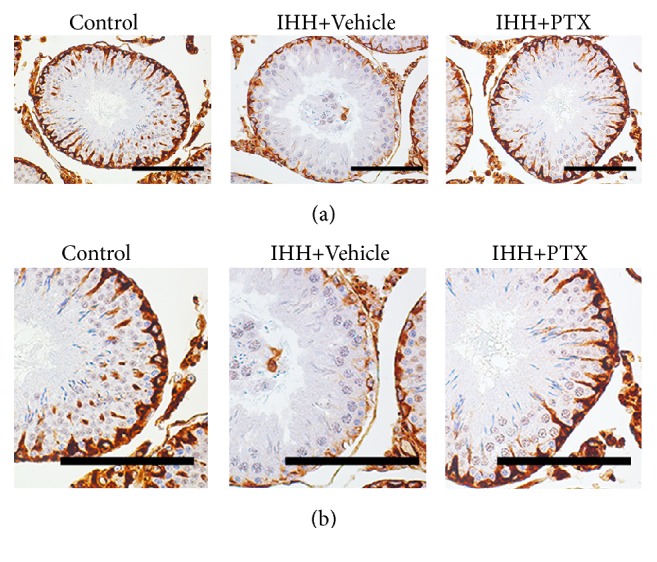
Immunohistochemical staining of vimentin in Sertoli cells. Lower panels represent a higher magnification of the corresponding upper ones. Scale bar = 100 *μ*m. Control group (*n* = 8); intermittent hypobaric hypoxia (IHH) group (*n* = 8); pentoxifylline (PTX)+IHH group (*n* = 8).

In Control animals, vimentin surrounded the nucleus and showed apical extensions in a “spoke-like” pattern in Sertoli cells from Control mice as previously observed [30]. IHH treatment reduced vimentin expression, accompanied by the disappearance of the spoke-like structure. Interestingly, PTX administration improved the morphological changes induced by IHH, enhanced vimentin expression, and reestablished the spoke wheel-like structure (Figure 6).

## 4. Discussion

PTX is one of the phosphodiesterase inhibitors reported to increase intracellular cyclic AMP and reduce superoxide anion production by both monocytes and polymorphonuclear cells dose dependently* in vitro* [26]. Endres et al. indicated that PTX causes a marked increase of cyclic AMP levels, whereas cyclic GMP levels are only marginally elevated in lipopolysaccharide stimulated human monocytes [27]. PTX has received considerable attention with respect to its action on leukocytes in many organs [28, 29, 31].

Previous studies have also confirmed the potential antioxidant effects of PTX [30, 32, 33]. Pozor et al. demonstrated that PTX may be a potential protective agent for preventing the negative changes related to oxidative stress in testicular injury caused by spermatic cord torsion in miniature horse stallion [34]. However, the protective effect of pentoxifylline against testis injury induced by intermittent hypobaric hypoxia is not established to date. In the current study, we tested the hypothesis that PTX could protect the testis from intermittent hypobaric hypoxia.

Oxidative stress is associated with an increased rate of cellular damage induced by oxygen and oxygen-derived oxidants, commonly known as reactive oxygen species [35, 36]. The major targets of reactive oxygen species are membrane lipids, in a process known as lipid peroxidation. It is also admitted that testicular tissues and spermatozoa are very sensitive to reactive oxygen species attack and lipid peroxidation. The susceptibility of testicular tissues to oxidation is attributed to the high polyunsaturated fatty acid content of sperm membranes [36, 37]. Many tissues contain powerful endogenous scavengers that provide protection against free radical damage, including SOD, CAT, glutathione peroxidase, ascorbic acid, and *α*-tocopherol [38]. Sikka et al. reported that adequate levels of antioxidants such as SOD and CAT, and possibly glutathione peroxidase and reductase, maintain the scavenging potential in gonads and seminal fluids, which is referred to as oxidative stress status [39]. CAT and SOD activity levels are increased during oxidative stress. In the present study, SOD levels and CAT activity were decreased significantly in the IHH+Vehicle group compared with the PTX+IHH group. Tissue MDA activity levels in testes were increased significantly in the IHH+Vehicle group compared with the PTX+IHH group. The above data regarding CAT and MPO activity levels and SOD amounts suggested a protective effect of PTX against testicular injuries. These data together with previous findings confirm the potent antioxidation capacity of PTX. Indeed, PTX possesses both antioxidative (oxidation prevention) and ROS scavenging (ROS identification and inhibition) properties [40]. These findings also support the notion that PTX may be an effective therapeutic adjunct to testis injury [41].

In this study, we found that epididymal sperm density and sperm mobility in IHH treated male rats were significantly decreased, accompanied with histopathologic changes in testicular tissues. These results demonstrated that IHH impaired spermatogenesis in male rats, corroborating previous reports [42–45].

The mechanisms of IHH which induced spermatogenesis impairment are not fully illustrated. It was reported that IHH could induce oxidative stress, which causes damage to testicular tissues. Our results revealed that IHH treatment increased the level of serum lipid peroxidation, which is an indicator of oxidative damage. Meanwhile, the antioxidant enzymes were significantly downregulated in testicular tissues. Together, these results indicated that oxidative stress plays an important role in the spermatogenesis dysfunction induced by IHH.

Spermatogenesis is a multifactor regulated process, where Sertoli cells play a crucial role. The latter cells reside in the basement membrane of seminiferous tubules and surround adjacent male germ cells. They provide physical and nutritional support to developing germ cells [46]. The tight junctions between Sertoli cells form a blood-testis barrier (BTB), which physically divides the seminiferous epithelium into basal and adluminal compartments. The BTB prevents the invasion of macromolecule to the seminiferous epithelium, creating a favorable microenvironment for spermatogenesis [40]. Furthermore, in mature testicular tissues the shrinkage of microfilaments and microtubules in Sertoli cells promotes the movement from basilar lamina to lumen of germ cells, which finally release the mature sperm to the lumen [47]. Moreover, plenty of germ cells undergo apoptosis during the developmental process. Sertoli cells can intake and degrade the apoptotic germ cells and their fragments [48]. This process plays important role in the maintenance of microenvironment. Therefore, the impairment of structure and function of Sertoli cells will significantly influence spermatogenesis.

Vimentin is an intermediate filament protein expressed in Sertoli cells and important constituent of microfilaments and microtubules. In addition, vimentin is involved in the apoptotic process of Sertoli cells [49]. It was reported that oral administration of di (n-butyl) phthalate impairs vimentin filaments of Sertoli cell and induces spermatogonium apoptosis [50]. Therefore, any factors impairing the vimentin filaments would further alter spermatogenesis. In this study, we observed that vimentin expression was reduced under IHH conditions. Meanwhile, the classical spoke-wheel-like structure of vimentin filaments from basal lamina to lumen disappeared. These results suggested that IHH condition may impair vimentin filaments by inducing oxidative stress in the testis, which is the underlying mechanism of IHH induced impairment of reproductive function in males.

PTX is a competitive nonselective phosphodiesterase inhibitor. It raises intracellular cAMP, activates PKA, reduces innate immunity and inflammation, and improves hemorheology [51]. Savas et al. reported that PTX treatment attenuates reperfusion damage by decreasing MDA levels and alleviating interstitial injury in rat testes [41]; PTX also improved the blood flow to both testes during detorsion, decreased germ cell apoptosis, and increased the total antioxidant capability to protect testes from the impairment caused by testicular torsion. In this study, PTX was applied to investigate its protective effect on the impairment caused by IHH treatment. As shown above, PTX administration decreased serum lipid peroxidation by increasing the activity of antioxidant enzymes and total antioxidant activity. PTX treatment also increased the expression of testosterone synthase and improved epididymal sperm density and mobility. Moreover, PTX treatment attenuated the IHH induced morphological changes in Sertoli cells as well as the histopathological alterations of the testicular tissue. Interestingly, reduced NADPH oxidase activity was observed in rats treated with PTX prior to iohexol used for kidney injury induction; the effects of PTX on O_2_^∙^ described in this study might be mediated through NADPH oxidase inhibition as well, which deserves further studies.

A limitation of this study is that it was an animal experimentation, and only a small number of rats were analyzed. Therefore, additional randomized, single, and multicenter trials with large sample size are warranted to confirm our results and generalize the clinical use of PTX. In addition, we did not assess whether PTX can play scavenging and/or AOX induction roles in Control animals. However, PTX alone was reported not to influence body weight, testis weight, serum testosterone level, and Johnsen's score in rats. Combined with previous findings, our results demonstrated that PTX can induce antioxidative activity under different conditions, including hind limb ischemia/reperfusion- and IHH- induced testicular injury.

## 5. Conclusion

IHH treatment significantly induced the peroxide status in animals and altered the normal function of spermatogenesis and testosterone synthesis. Oral administration of PTX significantly improved these pathological changes. Maintenance of redox homeostasis by PTX may be the underling mechanism.
